# Multiple Mitral Paravalvular Leak Closure With VSD Occluder in a High Surgical Risk Patient

**DOI:** 10.1016/j.jaccas.2026.107351

**Published:** 2026-03-14

**Authors:** Shubham Mandhare, Hemant Kokane, Niraj Pawar, Abhinav Mohabey, Anita Basavaraj, Haridas B. Prasad

**Affiliations:** a Department of Cardiology, Byramjee Jeejeebhoy Government Medical College & Sassoon General Hospital, Pune, India; b Department of General Medicine, Byramjee Jeejeebhoy Government Medical College & Sassoon General Hospital, Pune, India

**Keywords:** agilis sheath, KONAR-MF VSD device, mitral valve, paravalvular leak, transcatheter, transesophageal echocardiography

## Abstract

**Background:**

Mitral paravalvular leak (PVL) is a common complication after surgical mitral valve replacement, often causing symptomatic congestive cardiac failure. When transcatheter closure is planned, transesophageal echocardiography is essential for guiding percutaneous device closure.

**Case Summary:**

A 21-year-old woman with rheumatic heart disease and prior metallic mitral valve replacement presented with congestive cardiac failure. Transesophageal echocardiography revealed mitral paravalvular leaks and low ejection fraction. Considering her high surgical risk, the heart team opted for percutaneous PVL closure.

**Discussion:**

Mitral PVL after surgical mitral valve replacement can occur due to valve suture dehiscence. To our knowledge, we report the first case of multiple serpiginous mitral PVLs successfully closed in a single sitting, using 2 KONAR-MF VSD occluder devices, in a high surgical risk patient. The case highlights the flexibility and conformability of the KONAR-MF VSD occluder device, enhanced deliverability using a smaller sheath, and occlusive fabric promoting improved sealing. Tailored transcatheter closure can produce successful outcomes while minimizing the risk profile, in complex situations.

**Take-Home Message:**

Transcatheter closure of mitral PVLs with the KONAR-MF VSD occluder device is a safe and effective alternative to redo surgery in high surgical risk patients, providing symptomatic relief and improved cardiac function.

## Graphical abstract

**Figure:**
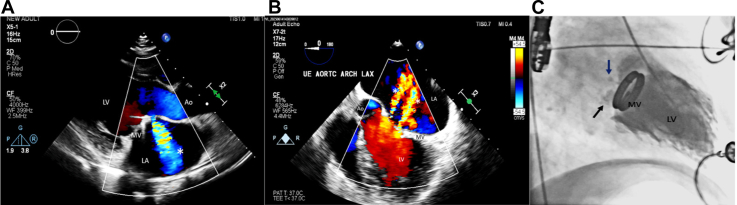

## Case Presentation and Past Medical History

A 21-year-old woman presented with exertional breathlessness (NYHA functional class III) for 3 months, progressing to orthopnea over 2 weeks. Six months earlier, she underwent mechanical mitral valve replacement with a 29-mm Chitra TTK mitral prosthesis (TTK Healthcare Ltd) for severe mitral stenosis secondary to rheumatic heart disease. She was diagnosed with congestive heart failure and showed clinical improvement after treatment with diuretics and vasodilators.

**Take-Home Message**

• Transcatheter closure of mitral PVLs with the KONAR-MF VSD occluder device is a safe and effective alternative to redo surgery in high surgical risk patients, providing symptomatic relief and improved cardiac function.

## Investigations

**Figure 1:**
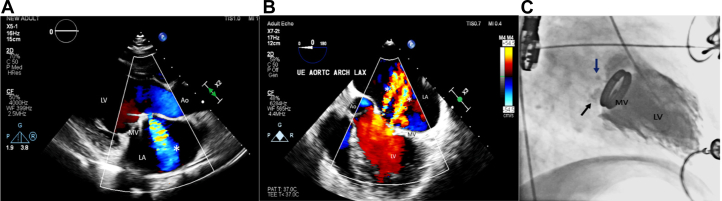
TTE, TEE, and Fluoroscopic Images (A-C): Preoperative transthoracic echocardiography (TTE), transesophageal echocardiography (TEE) images, and intraprocedural fluoroscopic images. (A) TTE parasternal long-axis view showing severe mitral regurgitation arising from aorto-mitral continuity. (B) TEE midesophageal 5-chamber view showing 2 paravalvular leak jets arising from aorto-mitral continuity (2 white asterisks). (C) Left ventricular angiogram (RAO 30° view) demonstrating 2 paravalvular leak jets (blue and black arrows). Ao = aorta; LA = left atrium; LV = left ventricle; MV = mitral valve; RAO = right anterior oblique.

Laboratory investigations showed elevated N-terminal pro–B-type natriuretic peptide levels with negative inflammatory markers. Transthoracic echocardiography revealed severe mitral regurgitation secondary to paravalvular leak (PVL) (color flow jet area: >60%, vena contracta: 7.0 mm, regurgitant fraction: 55%, regurgitant volume: 70 mL) and severe global left ventricular dysfunction (ejection fraction: 20%) with left atrial dilation (Figure 1A). Prosthetic valve gradients and leaflet excursion were normal.

**Figure 2:**
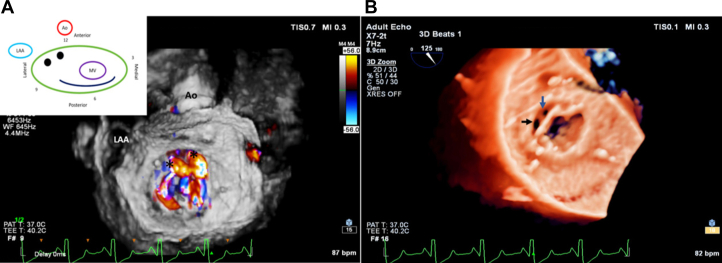
TEE Images of the Mitral Valve and Paravalvular Leaks (A and B) 3-dimensional TEE reconstruction images depicting surgeon's view of the mitral valve. (A) Surgeon's view of the mitral valve, represented by the clock face diagram showing 2 paravalvular defects at 10 o’clock and 12 o’clock positions. (B) The paravalvular defects are indicated by the blue and black arrows. LAA = left atrial appendage; other abbreviations as in Figure 1.

Transesophageal echocardiography (TEE) further characterized 2 paravalvular defects measuring 12.0 × 8.0 and 9.0 × 5.0 mm at the 10° and 12° clock positions, respectively (Figures 1B and 2).

Because of the high operative risk from severe left ventricular dysfunction, retrosternal adhesions from the previous sternotomy, and the requirement for ongoing anticoagulation, the heart team planned transcatheter PVL device closure.

## Management

**Figure 3:**
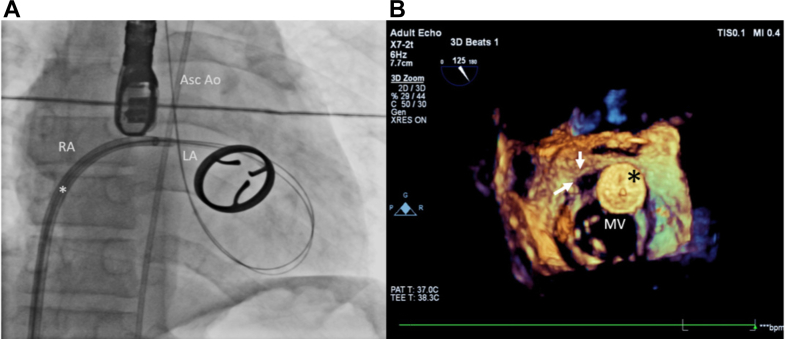
Intraoperative Fluoroscopic and TEE Images (A and B) Intraoperative fluoroscopic and TEE images. (A) Fluoroscopic AP view. The 0.032-in straight-tip Terumo wires are being delivered through the Agilis steerable sheath (white asterisk) across both paravalvular leaks. (B) 3-dimensional TEE reconstructed image of the first KONAR-MF VSD occluder device (black asterisk) after deployment. The arrows represent the adjacent persisting paravalvular defect. AP = anteroposterior; Asc Ao = ascending aorta; RA = right atrium; other abbreviations as in Figure 1.

Transcatheter PVL device closure was performed under general anesthesia and mechanical ventilation. After securing bilateral femoral arterial (6-F) and right femoral venous (6-F) accesses, left ventricular angiograms were obtained in the right anterior oblique (30°) and left anterior oblique (30°) views (Figure 1C, Video 1). The venous access was upgraded to an 8.5-F 71-cm Agilis NxT steerable sheath (St Jude Medical). Under 3-dimensional TEE guidance, transseptal puncture was performed, and the Agilis sheath was advanced into the left atrium. Two straight-tip 260-cm 0.032-in Terumo soft wires (Terumo Corp) were advanced through both paravalvular defects (Figure 3A, Video 2) under 3-dimensional TEE guidance, then exchanged with 2 Amplatz superstiff wires using Judkins right catheters. Thickening and extensive fibrosis of the interatrial septum caused tenting and difficulty in transseptal puncture, necessitating the use of superstiff wires for crossing the PVLs, in anticipation of further procedural difficulties.

The first arteriovenous loop was established through the left femoral arterial sheath. Then, a 7-F patent ductus arteriosus delivery system was advanced through the left atrium and across the defect into the ascending aorta.

**Figure 4:**
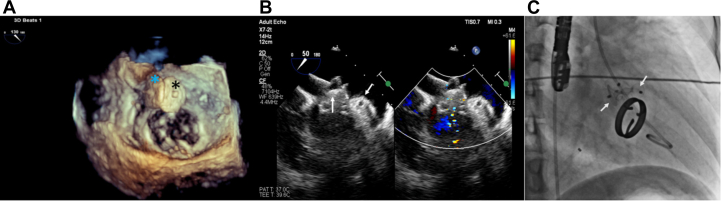
Post-Deployment TEE and Fluoroscopic Images (A to C): Post-deployment transesophageal echocardiography (TEE) and fluoroscopic images. (A) Both the KONAR-MF VSD occluder devices (blue and black asterisks) are shown. (B) TEE midesophageal 50° view with color Doppler, indicating absence of paravalvular leak. Arrows indicate the positions of both paravalvular devices. (C) Fluoroscopic image (RAO 30° view) of both KONAR-MF VSD occluder devices.

A 14- × 10-mm KONAR-MF VSD occluder device (Lifetech Scientific) was deployed across the 12- × 8-mm paravalvular defect (Figure 3B, Video 3), and released after confirming optimal positioning. After deployment of the first device, a second arteriovenous loop was created through the left femoral arterial access, and the 7-F patent ductus arteriosus delivery sheath was advanced. A second KONAR-MF VSD occluder device measuring 12 × 10 mm was deployed through the 9- × 5-mm defect (Figure 4A, Videos 4 and 5). Post-deployment imaging showed no residual leak, no left ventricular outflow tract obstruction, and decreased prosthetic mitral leaflet movement (Figures 4B and 4C). The patient remained hemodynamically stable and was successfully extubated.

## Patient Outcome

Postoperatively, the patient received oral vitamin K antagonists after heparin bridging along with aspirin. At 1-month follow-up, she remained hemodynamically stable and asymptomatic. Echocardiography showed no residual leak with devices in situ and a significant improvement in left ventricular ejection fraction to 45%.

## Discussion

Mitral PVL remains a challenging complication after surgical mitral valve replacement, with an incidence of 7% to 17% depending on the valve type and surgical technique. Anatomically, the mitral annulus is nonuniform—its posterior annulus is longer and less fibrous, increasing susceptibility to mechanical stress and suture dehiscence. This predisposes the posteromedial and anterolateral segments of mitral annulus to PVL formation. Furthermore, postoperative annular dynamics in the form of annular rigidification and abnormal systolic tilt further exacerbate leak formation.^1^^,^^2^ Surgical reoperation carries high perioperative risk, particularly patients with ventricular dysfunction, retrosternal adhesions, or multiple comorbidities, making minimally invasive transcatheter closure an attractive and safer alternative.^3^^,^^4^ The transcatheter approach offers the advantage of lower short-term mortality and reduced hospital stay compared with reoperation. Although it may be associated with higher rates of residual leaks and recurrent symptoms, experienced centers achieve technical success rates of >85%, resulting in symptomatic improvement for most patients.^5^^,^^6^ The tortuous, elliptical, or crescentic shape of PVLs, in concert with catheter crossing and device positioning, can produce significant procedural difficulties.^7^^,^^8^ The steerable Agilis sheath enabled the navigation of multiple stiff wires across the paravalvular defects.

**Figure 5:**
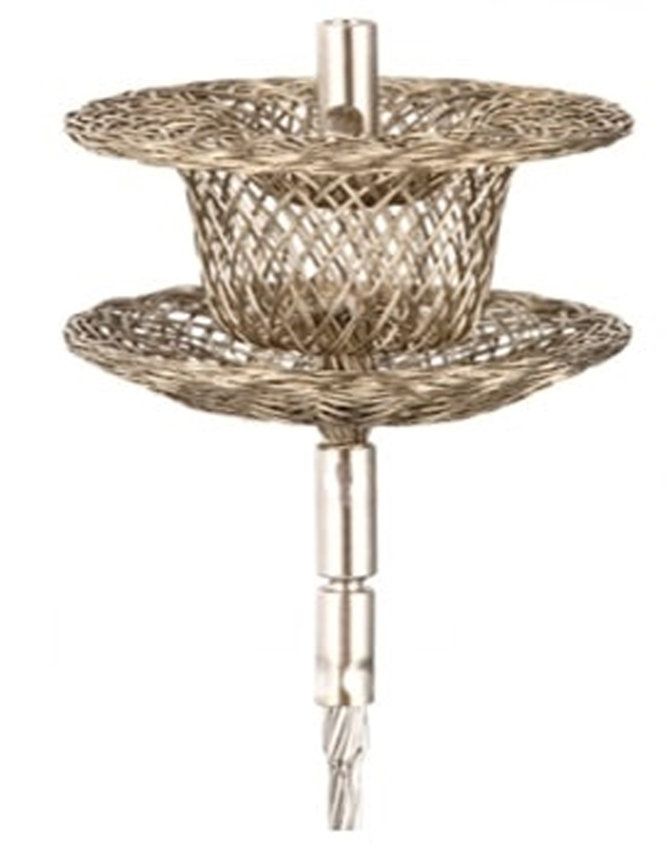
The KONAR-MF VSD Occluder Device KONAR-MF VSD occluder device (Picture credits: Lifetech Scientific, China).^9^

Device selection is critical because no single closure device is ideal for all PVLs. The Amplatzer Vascular Plug-III is widely used because its elliptical dual-disc design adapts well to crescentic defects and minimizes prosthetic leaflet interference.^8^ The KONAR-MF VSD occluder (Figure 5) offers superior versatility due to its self-expandable, flexible, double-disc configuration and lower profile delivery system (4- to 7-F). This enables better navigation through complex anatomies and serpiginous leaks.^9^^,^^10^ The fabric component of the KONAR-MF VSD occluder may enhance complete sealing by promoting tissue ingrowth and occlusion. Its radial force allows the device to conform to the shape of the defect, losing its self-centering property. The dual-screw delivery system facilitates both antegrade and retrograde deployment approaches, which is advantageous in challenging anatomies.^10^^,^^11^ We hypothesize that the alignment of both devices will lead to enhanced stability and defect sealing after endothelization. Despite encouraging outcomes, complications such as hemolysis can occur, particularly with residual or incomplete sealing. Careful assessment for device interference with prosthetic valve leaflets is essential.^6^^,^^7^ Long-term data confirm that achieving successful closure (to mild or less regurgitation) correlates with improved survival and fewer heart failure hospitalizations.^12^ Our experience supports transcatheter closure as a first-line therapy for high-risk surgical candidates. Early intervention before the onset of ventricular dysfunction may optimize recovery potential. The KONAR-MF VSD occluder device and Agilis sheath represent valuable additions to the armamentarium for complex mitral PVL closure due to their superior design, maneuverability, and deliverability. This merits further evaluation in large-scale prospective studies.

## Conclusions

This case demonstrates the feasibility and effectiveness of using the KONAR-MF VSD occluder for transcatheter closure of complex mitral PVLs in high-risk surgical patients. Early intervention with a flexible, anatomically adaptable device can achieve complete sealing, leading to significant clinical improvement and left ventricular functional recovery while avoiding redo surgery. Multidisciplinary evaluation and individualized procedural planning remain critical to optimize outcomes. Future prospective studies are necessary to define long-term device durability and comparative efficacy against surgical approaches.

## Funding Support and Author Disclosures

The authors have reported that they have no relationships relevant to the contents of this paper to disclose.

## Appendix

Video 1 Left Ventricular Angiogram (right anterior oblique 30° View) Demonstrating Both Paravalvular Leaks at 9 O’clock and 10 O’clock Positions, and the Prosthetic Mitral Valve

Video 2 Fluoroscopic AP View Showing the Arteriovenous Loop Across Both Paravalvular Defects With the Help of the Agilis Sheath

Video 3 Fluoroscopic AP View and Deployment of the First KONAR-MF VSD Occluder Device

Video 4 Left Ventricular Angiogram (RAO 30°) View After Positioning of the Second Occluder Device, Showing Absent Flow Across Both Devices

Video 5 Fluoroscopy at the Time of Releasing the Second Occluder Device
